# Automated Analysis of Craniofacial Morphology Using Magnetic Resonance Images

**DOI:** 10.1371/journal.pone.0020241

**Published:** 2011-05-31

**Authors:** M. Mallar Chakravarty, Rosanne Aleong, Gabriel Leonard, Michel Perron, G. Bruce Pike, Louis Richer, Suzanne Veillette, Zdenka Pausova, Tomáš Paus

**Affiliations:** 1 Rotman Research Institute, Baycrest, Toronto, Ontario, Canada; 2 Mouse Imaging Centre (MICe), The Hospital for Sick Children, Toronto, Ontario, Canada; 3 Montréal Neurological Institute, McGill University, Montréal, Québec, Canada; 4 ÉCOBES, Recherche et Transfert, Cégep de Jonquière, Jonquière, Québec, Canada; 5 Université du Québec à Chicoutimi, Saguenay, Québec, Canada; 6 Département des Sciences de l'Éducation et de Psychologie, Université du Québec à Chicoutimi, Chicoutimi, Québec, Canada; 7 School of Psychology, University of Nottingham, Nottingham, United Kingdom; 8 The Hospital for Sick Children, Toronto, Ontario, Canada; 9 Centre de Recherche, Centre Hospitalier de l'Université de Montréal, Montréal, Québec, Canada; Institute of Automation, Chinese Academy of Sciences, China

## Abstract

Quantitative analysis of craniofacial morphology is of interest to scholars working in a wide variety of disciplines, such as anthropology, developmental biology, and medicine. T1-weighted (anatomical) magnetic resonance images (MRI) provide excellent contrast between soft tissues. Given its three-dimensional nature, MRI represents an ideal imaging modality for the analysis of craniofacial structure in living individuals. Here we describe how T1-weighted MR images, acquired to examine brain anatomy, can also be used to analyze facial features. Using a sample of typically developing adolescents from the Saguenay Youth Study (N = 597; 292 male, 305 female, ages: 12 to 18 years), we quantified inter-individual variations in craniofacial structure in two ways. First, we adapted existing nonlinear registration-based morphological techniques to generate iteratively a group-wise population average of craniofacial features. The nonlinear transformations were used to map the craniofacial structure of each individual to the population average. Using voxel-wise measures of expansion and contraction, we then examined the effects of sex and age on inter-individual variations in facial features. Second, we employed a landmark-based approach to quantify variations in face surfaces. This approach involves: (a) placing 56 landmarks (forehead, nose, lips, jaw-line, cheekbones, and eyes) on a surface representation of the MRI-based group average; (b) warping the landmarks to the individual faces using the inverse nonlinear transformation estimated for each person; and (3) using a principal components analysis (PCA) of the warped landmarks to identify facial features (i.e. clusters of landmarks) that vary in our sample in a correlated fashion. As with the voxel-wise analysis of the deformation fields, we examined the effects of sex and age on the PCA-derived spatial relationships between facial features. Both methods demonstrated significant sexual dimorphism in craniofacial structure in areas such as the chin, mandible, lips, and nose.

## Introduction

Anthropologists have long analyzed craniofacial features on skull remains obtained from hominids, Neanderthals, apes and modern humans to study differences between species, early migration patterns, and phenotypic differences within Neanderthals. For example, analyses of mandibular [1] and cranial [2] structure have helped scholars understand the morphometric signatures specific to different Neanderthals, in comparison with modern humans and different subspecies of chimpanzees. Further, by quantifying differences and similarities in craniofacial structure, these studies have helped refine theories regarding the evolutionary lineages of specific classes of Neanderthals [2], [3] and hominids [1], and settlement patterns of modern humans [4], [5].

Biomedical research has recently started using craniofacial structure to examine specific phenotypes in the context of brain dysfunction, hormonal environments, and sexual dimorphism. For example, Cohen *et al*. [6] demonstrated a divergence in craniofacial structure early in fetal life when comparing fetuses with Down's syndrome with healthy ones. Several groups have shown that patients suffering from schizophrenia have characteristic craniofacial phenotypes that include elongation of the craniofacial structure [7], [8] and sexually dimorphic asymmetries [9]. Hennessey *et al*. [10] demonstrated frontonasal dysmorphologies, such as increased width of the nose, narrowing of the mouth, and upward displacement of the chin, as being specific to patients suffering from bipolar disorder. Sexually dimorphic characteristics in the mouth and chin structure have been demonstrated in normal young adults [11]. There is also evidence of a relationship between the ratio of the lengths of the second and fourth digits (2D:4D ratio; a surrogate marker of prenatal testosterone exposure) and craniofacial structure, such as a broadening of the mandible and zygomatic arch with a decreased digit ratio [12], [13].

Morphometry-based classification of skull remains is generally performed using the variability in the position of landmarks and semi-landmarks, the latter being landmarks defined in relation to precise craniofacial features [2], [4]. In human biomedical research, similar point-based methodologies have been used. These studies use landmarks defined on photographs [12], [13], surfaces created through advanced laser-scanning techniques [9], [10], [11], [14], or whole-head magnetic resonance images (MRI) [8], [15]. In general, most research groups use a similar computational methodology to determine variations in craniofacial morphometry, namely point-distribution models (PDM) [16]. The variability in the size and shape of a face is accounted for by transforming each individual to the average dimensions of the entire population under study, i.e. the *Procrustes superposition* of all landmarks, such that all landmark positions are transformed to a common coordinate space and reflect the remaining nonlinear differences in the population [17], [18]. The actual coordinate locations for each of the landmarks in this space are analyzed to determine the variability of craniofacial features.

The current work is motivated by the availability of MR images collected in a number of large neuroimaging initiatives [19], [20], [21], the abundance of state-of-the-art techniques for image processing of brain MR images [22], [23], readily available statistical techniques for voxel-wise analyses [24], and the research interest in craniofacial morphology reviewed above. Here, we use T1-weighted data from the Saguenay Youth Study [25] to analyze the sexual dimorphism and age-related changes in the morphometry of the adolescent face. Our main goal is to demonstrate how T1-weighted MR images can be used in both the voxel-wise analysis of facial features and the decomposition of facial features using principal components analysis (PCA). The voxel-wise analysis borrows from a group-wise deformation-based analysis of brain MRI data [26], [27], [28] and requires the creation of an average model of the face in the population; the deformations that map each individual to the average model are then used to quantify group-wise differences in facial features. The second analysis is an extension of anthropometric studies conducted both in two and three dimensions [11], [12], [13] and relies on the decomposition of variances in landmark-based data.

First, we demonstrate the methods for the development of a population-based model using nonlinear registration and the voxel-by-voxel analyses of the deformation data. In a second analysis, we use the model and nonlinear transformations to analyze facial shape using landmark-based data.

## Methods

### Participants

All participants are white Caucasians recruited from a population with a known genetic founder effect living in the Saguenay Lac Saint-Jean (SLSJ) region of Quebec, Canada. The MR images have been acquired in the context of the Saguenay Youth Study (SYS), which is described in detail in Pausova *et al*. [25]. Briefly, participants were recruited in secondary schools in the SLSJ region. A research nurse conducted a telephone interview with interested families (usually with the child's mother) to verify their eligibility. Additional information was acquired using a medical questionnaire completed by the child's biological parent. The main exclusion criteria were as follows: (1) positive history of alcohol abuse during pregnancy; (2) positive medical history for meningitis, malignancy, and heart disease requiring heart surgery; (3) severe mental illness (e.g., autism, schizophrenia) or mental retardation (IQ<70); and (4) MR contraindications. At the time of the analysis, data from 621 participants (12 to 18 years of age) were available. All participants filled out the Puberty Development Scale (PDS), which is an eight-item self-report measure of physical development based on the Tanner stages with separate forms for males and females [29]. There are five categories for this scale of pubertal status: (1) prepubertal, (2) beginning pubertal, (3) midpubertal, (4) advanced pubertal, and (5) postpubertal (see also [25], [30], [31]). Twenty-four adolescents were excluded from the study as they had orthodontic work that resulted in large-scale image artefacts in the imaging data. This left a cohort of 597 adolescents (292 male, 305 female; see Figure S1 for graphical distribution by age). A demographic summary of the study participants, including sex, age, full-scale intelligence quotient, and pubertal stage is given in Table 1.

**Table 1:** Demographic summary of the adolescent participants.

	*Males*	*Females*
***Total participants***	292	305
***Age (in months)***	180.5 (22.2)	181.8 (23.0)
***Full-scale IQ***	104.3 (14.6)	104.2 (13.2)
***Puberty Stage***	3.4 (0.9)	4.1 (0.7)

Values are given as the mean (standard deviation) where applicable.

Ethics approval for data collection from the adolescents who participated in this study was provided by the research ethics committee from the Centre de santé et de services sociaux de Chicoutimi. All participants in the study provided informed written assent for this study and their parents provided informed written consent for the inclusion of their child in this study.

### T1-weighted MRI

For each participant, T1-weighted MR images of the brain were acquired on a Philips 1.0-T superconducting magnet using the following parameters: three-dimensional (3D) radio frequency (RF)- spoiled gradient-echo scan with 140 –160 slices, an isotropic resolution of 1 mm, a repetition time (TR) of 25 ms, an echo time (TE) of 5 ms, and flip angle of 30°.

### Creation of a minimally biased model and voxel-wise analyses

#### Data analysis/Image processing

**Figure 1:**
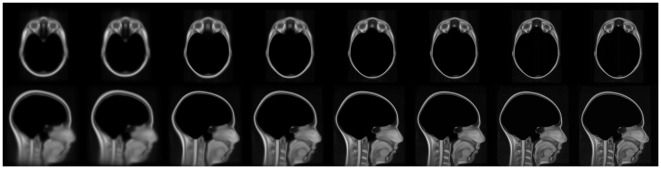
Population averages at each iteration in the hierarchical model building process. For each step in the model-building process, axial (top row) and sagittal (bottom row) views are shown. From left to right: The 9-parameter linear, 12-parameter linear, and each of the 6 nonlinear models (from each step outlined in Table 1). Note the improved contrast and structural resolution at each step in the model building process.

In order to estimate differences in shape between faces within the population, a group-wise nonlinear average of the craniofacial features was estimated using methods similar to those used in the deformation-based analysis of brain anatomy in humans [32] and animals [33], [34], [35], [36]. All scans were first corrected for intensity inhomogeneity using the N3 algorithm [37]. To initialize the model building process, a single T1-weighted MRI was randomly chosen from the sample to be the target for all other image volumes. All other MRI volumes were then rigidly rotated and translated (3 rotations and 3 translations) to match this initial target. The brain was then extracted using the “Brain Extraction Tool” [38], leaving only craniofacial information in each of the images. The remaining data includes skull (including teeth) and soft tissue (skin, muscle, and subcutaneous fat), thereby allowing for the analysis of craniofacial features with respect to the composite of tissue types from which the features are created. We estimate nonlinear transformations based on local intensity information. This method should mitigate the inclusion of such information as the teeth. As a result of the brain extraction, the following linear and nonlinear registration steps are driven only by intensity information in craniofacial structures. All possible pair-wise 9-parameter transformations (3 rotations, 3 translations, and 3 scales; 596 transformations for each of the 597 participants) were estimated and an average linear transformation was calculated for each image, thus effectively scaling each individual scan to the average head and face size of the population. After applying the average transformation, scans were averaged and the original scans were registered to this model using a 12-parameter transformation (3 rotations, 3 translations, 3 scales, and 3 shears); a new population-based average was estimated at this point. This model represents the population model accounting for all linear differences in head size. A multi-generation, multi-resolution fitting strategy was then initialized where each head was nonlinearly registered to the 12-parameter population atlas and another population-based average was estimated at this point. The group-wise atlas is generated in this iterative fashion, where all heads are nonlinearly registered to the atlas of the previous nonlinear registration using nonlinear transformations of increasing resolution at each iteration. The resulting transformations map the craniofacial structure of each individual to the nonlinear average of the entire group and can be analyzed explicitly to determine local variations in shape. Linear [39] and nonlinear [40] transformations were estimated using the mni_autoreg package available as part of the MINC toolbox (http://packages.bic.mni.mcgill.ca/). Nonlinear transformations were estimated using the previously optimized version of the ANIMAL algorithm [41]. Table 2 contains the parameters used at each stage of the nonlinear model-building process. Figure 1 demonstrates the results of the population averaging at each iteration in the model-building process.

**Table 2:** Listing of the registration parameters used in the nonlinear model-building process.

*Step size (mm)*	*Iterations*	*Gaussian Blur (mm)*
8	30	16
8	30	8
4	30	8
4	30	4
2	10	4
2	10	2

At each stage, the intensity-blurred images were matched to one another. A 3D simplex optimization was used with stiffness, weight, similarity parameters set to 1, 1, and 0.3 respectively (as optimized in [41]). In each case the spherical search area around each node was set to *3 x step size*.

Voxel-wise analysis of deformation fields. Shape differences were analyzed over the entire extent of the craniofacial region but excluding the parts of the head posterior to the top of the forehead, thus limiting the analysis to variations in facial morphometry. The Jacobian determinants [23], providing an index of local volume expansion or contraction, were computed at every voxel. Each Jacobian-determinant map was blurred using a Gaussian kernel with an 8-mm full-width at half-maximum. The statistical analysis was carried out with the *fmristat* (http://www.math.mcgill.ca/keith/fmristat/) software packages and multiple comparisons were corrected using Gaussian Random Field Theory (*p<0.05*, corrected).

The voxel-wise analyses were carried out to examine the effect of sex while covarying for age and overall head size (derived from the multiplication of the three scaling factors estimated for each subject; a standard procedure employed in many morphological neuroimaging studies). To analyze the effect of age, we carried out separate analyses in male and female adolescents while covarying for overall headsize.

### Landmark-based facial feature analysis

While the above deformation-based analysis gives a measure of local expansions and contractions, it does not provide an intuitive representation of the actual facial features and their shape. To analyze these relationships, we draw from previous work in our group on morphing body-images [42] for examining differences in visual body perception [43] and on the work of Fink *et al*. [12] who have used 2D photographs of faces of young adults to analyze the shape of the face. In what follows, we describe the development and analysis of a point distribution analyzed using Principal Component Analysis (PCA).

#### Identification of facial features using landmarks

**Figure 2:**
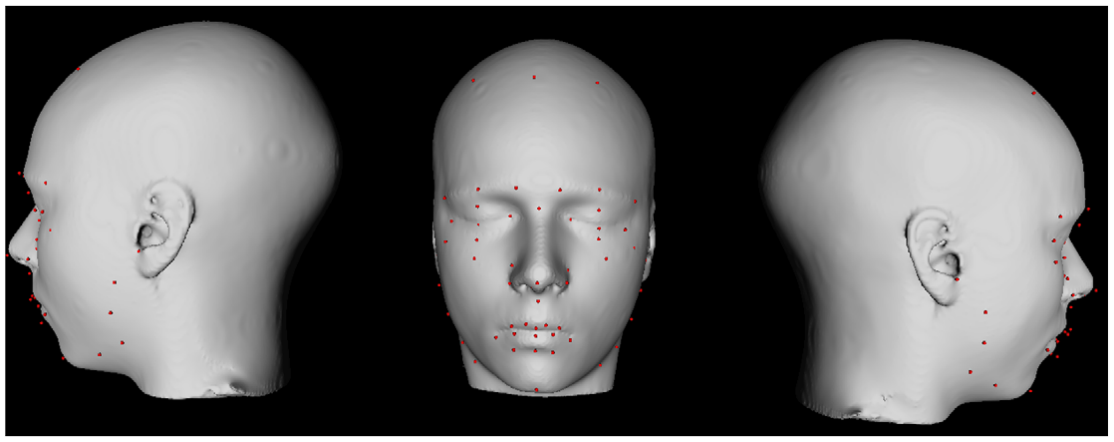
Facial landmarks placed manually on a surface-based representation of the population-based atlas. Landmarks are defined in red.

In order to create a point distribution, we use methods employed previously in model-based segmentation techniques in neuroimaging studies. In these types of methodologies [40], [44], anatomical landmarks are defined on an individual model and then warped back to individual subjects using a nonlinear transformation. In this case, two of the authors familiar with craniofacial anatomy (MMC and RA) placed landmarks on a surface- and voxel-representation of the nonlinear model defined in the previous section (see Figure 2). Our methods improve on this technique as landmarks need to be defined only on the model and are automatically customized to each individual face using the inverse of each individual's nonlinear transformation estimated previously (See 2.3.1). Note that this transformation brings the landmarks to the space corresponding to the linear (12-parameter) registration; as such, global differences in head size have been removed. This is analogous to the Procrustes method of superposition used in previous studies [12], [13].

Here we placed, on the average non-linear model, 56 landmarks similar to those employed by Fink et al. [12]. Most landmarks were defined using explicit anatomical definitions. A subset of these (as in [12], [42]) can be considered semi-landmarks defined by their relative position between landmarks. For example, a *semi-landmark* around the jaw was defined as the point located half the distance between the inferior tip of the chin and the maxillary process along the convexity of the jaw line. See Table S1 for a full listing of all landmarks used.

Accuracy of group-wise nonlinear registration. To evaluate the accuracy of the group-wise nonlinear registration strategy, we warped the landmarks defined using the inverse of the transformation that maps their craniofacial features to the model. A co-author on this paper (RA) manually identified 17 of the craniofacial landmarks on 10 randomly-selected subjects (see Table 3). All landmarks were identified using only information available in a tri-planar view using the Display software package (http://www.bic.mni.mcgill.ca/ServicesSoftwareVisualization/HomePage). Only full landmarks were chosen for this purpose. Accuracy and precision of the nonlinear transformations were evaluated by calculating the Euclidean distance between the homologous automatically and manually derived landmarks.

**Table 3:** Results from the comparison of warped landmarks to manually derived landmarks on ten subjects.

*Landmark*	*Label*	*Distance (mm)*	*Standard Deviation (mm)*
*10*	Lateral right eye	10.43	1.37
*11*	Medial right eye	10.13	1.18
*15*	Medial left eye	10.80	2.34
*16*	Lateral left eye	10.10	2.35
*20*	Middle of the base of nose	1.76	0.79
*21*	Tip of the nose	4.20	2.55
*22*	Bridge of the nose	2.47	0.71
*25*	Mid right nostril	2.56	1.24
*26*	Mid left nostril	2.97	1.29
*32*	Inferior Peak of the Midpoint of the Upper Lip	5.23	0.70
*37*	Inferior Peak of the Lower Lip	2.78	1.21
*38*	Left Mid-Lower Lip	10.33	0.89
*39*	Right Mid-Mouth Seam	2.58	1.00
*40*	Mid-Mouth Seam	1.88	1.02
*42*	Right ear	2.20	0.81
*43*	Left ear	1.58	0.62
*44*	Bottom of Chin	4.94	2.61

#### Anthropmetric analysis

As is often done in classical anthropometric studies, we also analyzed distances between landmarks. Here we chose a number of absolute distances, including the width and height of the left and right eyes, mouth width, the distance between the ears and the zygomatic arches, nose width, filtrum length (bottom of the nose to top of the lip), nose-to-chin length, and lip-to-chin length. See Table 4 for a full description of the distances analyzed. The absolute lengths were used to evaluate the effect of sex, age, and interactions of age and sex (4 degrees of freedom). All statistical analyses were performed in JMP8 (SAS; Cary, North Carolina, USA).

**Table 4:** Analysis of anthropometrics.

*Craniofacial structure*	*Landmarks*	*Sex*	*Age*	Age*Sex
Left eye length	15,16	7.12 ^***^	−1.37	1.33
Right eye length	10,11	3.76 ^**^	1.53	0.43
Left eye height	17,19	4.57 ^***^	−1.72	2.00 ^*^
Right eye height	12,14	5.67 ^***^	−1.13	2.52 ^*^
Mouth width	29,38	−9.77 ^***^	8.49 ^***^	5.63 ^***^
Craniofacial width (ear to ear)	41,43	−10.09 ^***^	0.90	−3.74 ^**^
Craniofacial width (zygomatic arch)	53,55	4.83 ^***^	0.56	−0.99
Nose width	23,24	−10.88 ^***^	3.39 ^**^	−5.22 ^***^
Filtrum (nose to upper lip)	20,32	−7.74 ^***^	1.70	−2.50 ^*^
Nose to tip of chin	20,44	−7.52 ^***^	5.33 ^***^	−3.01 ^*^
Bottom lip to tip of chin	37,44	−2.75 ^**^	5.59 ^***^	−2.28 ^*^

*p<0.05, **p<0.001, ***p<0.0001

For a full description of the landmark numbers, see Table S1. In each case, results are summarized as the linear model coefficients (values greater than 0 for sex indicate greater values in females).

#### Point-distribution model

To characterize the shape of the face, and to reduce the dimensionality of our landmark-based data, we developed a point-distribution model (PDM) [16]. PDMs have been used extensively in medical imaging; they rely on the assumption that variations in the shape of an object can be estimated reliably by modeling the spatial distribution of a series of appropriately placed homologous landmarks. Our PDM was computed using a PCA of the warped landmarks. The PDM was created using all three dimensions (x, y and z) from all 56 landmarks in 597 subjects. PCA was conducted using the *R* software package (http://www.r-project.org/). To quantify variability with respect to the original landmark locations, original x, y and z positions from the landmarked model were first subtracted from each coordinate point and the PCA was performed on the normalized coordinates.

#### Analysis of the principal components

Principal component (PC) scores were estimated for each individual and used to evaluate the effect of sex, age, and interactions of age and sex (4 degrees of freedom) for each of the first five PCs. All statistical analyses were performed in JMP8.

For visualization of the relationship between facial features captured in a single PC, we performed simulations for the first 5 PCs. Original landmarks (see Figure 2; Table S1) were displaced by adding a proportion of each component score (0.2, 0.4, 0.6, 0.8, and 1). A smooth three-dimensional warp matching the original landmarks to the displaced landmarks was defined using a thin-plate spline [45], [46].

## Results

### Voxel-by-voxel analysis of deformation fields

**Figure 3:**
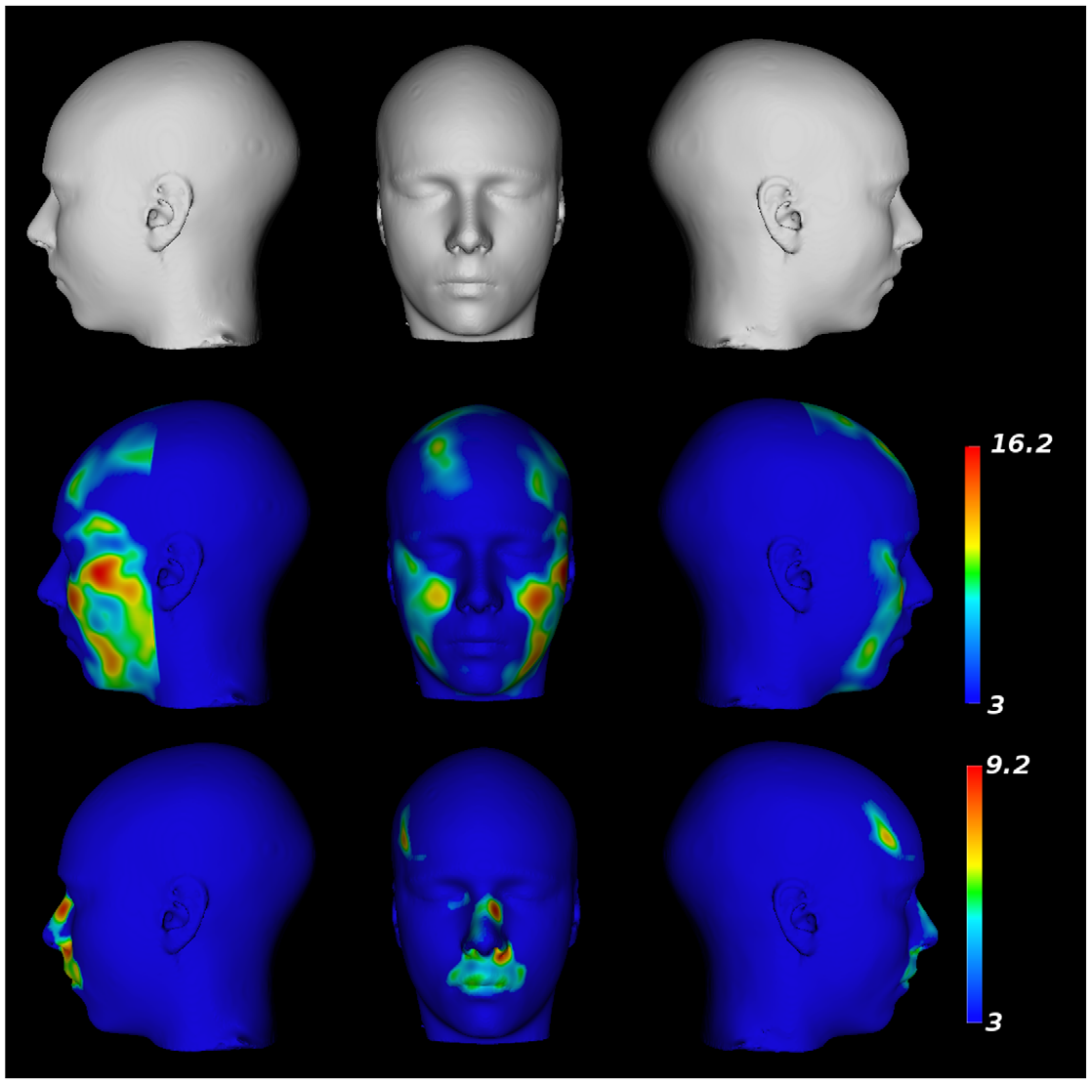
Results from the voxel-by-voxel analysis of deformation fields. Top Row: A surface rendered version of the population-based atlas. Middle Row: Parametric map projected onto the surface showing regions yielding statistically larger expansions in males in comparison to females. Bottom Row: Parametric map projected onto the surface showing regions yielding statistically larger expansions in females in comparison to males.

The results of the population-based model-building process demonstrate excellent alignment of the craniofacial structures. Figure 1 demonstrates axial and sagittal views from the model-building process. Each step in the process demonstrates increased structural contrast and anatomical resolution in comparison with the previous step. The initial population averages, generated by the 9-parameter and 12-parameter linear registrations, demonstrate large variability in the areas of the nose, chin, and lips. As expected, this variability is reduced considerably through each of the subsequent nonlinear steps. After visual inspection we determined that there were 28 overall registration failures during the image processing stages of the analyses. These subjects have been removed from the analysis. All further results are reported with these subjects removed. A surface-based representation (using a modified marching cubes-based extraction [47] of a segmentation of the final nonlinear model) is shown in the first row of Figure 3.

A voxel-wise analysis of sex differences (age removed) in the deformation fields demonstrates “expansion” of the mandible, chin, forehead, and zygomatic area in males in comparison with females. As seen in the middle row of Figure 3, this finding is represented as one continuous cluster within the search area (DF = 593, p = 4.0*×*10^−7^, cluster volume *[v]* = 6.80*×*10^5^ *mm^3^*, peak t-value  = 16.2). Females, as compared with males, show a far more localized expansion in the region of the lips, in the region between the lips and the nose, around the bridge of the nose, and near the left temple. These sex differences (females>males) are demonstrated in two different clusters; the first is a continuous cluster showing expansion of the lips, upper lip, and the bridge of the nose area (DF = 565, p = 4.0*×*10^−7^, *v = *5.68×10^5^ *mm^3^*, peak t-value  = 9.2) and the second is a smaller region in the left temple (DF = 565, p = 4.0*×*10^−7^, *v = *8.7×10^4^ *mm^3^*, peak t-value  = 8.9).

**Figure 4:**
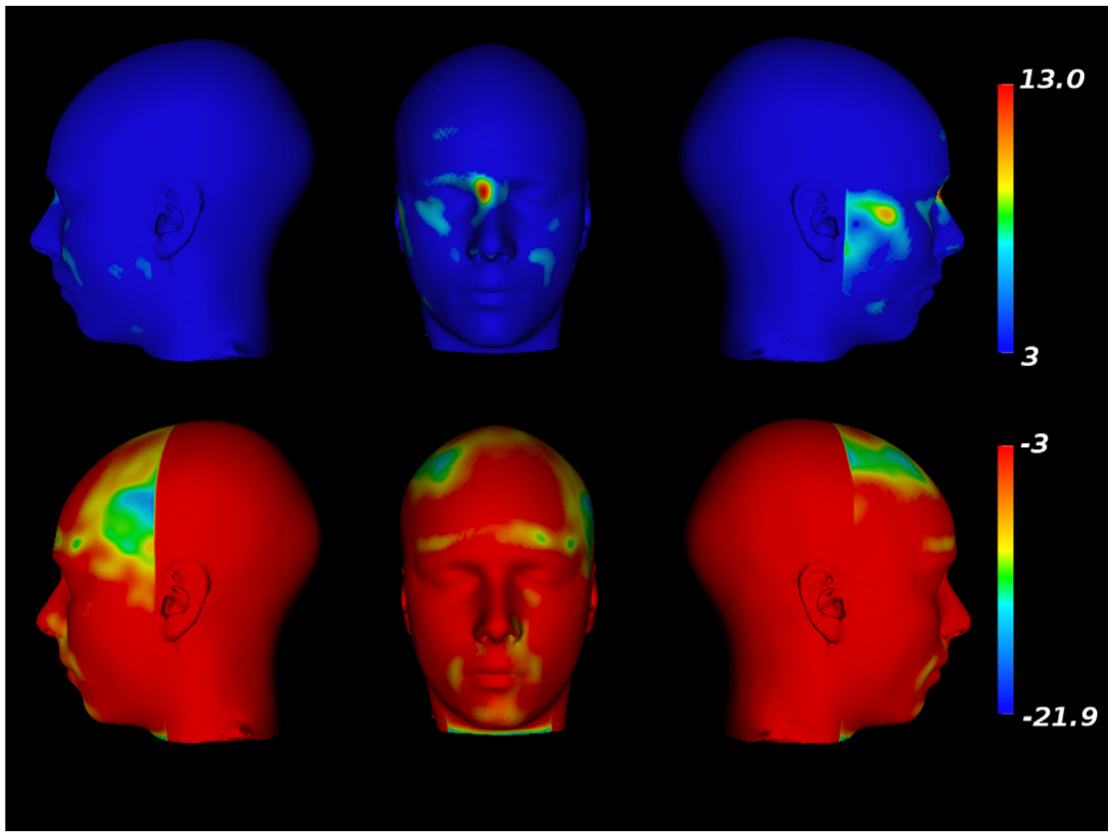
Facial morphometry changes related to age in males. Top row: Facial expansions related to age. Bottom row: Facial contractions related to age.

Voxel-wise analysis of age-related changes in the deformation fields, carried out separately for male and female adolescents, yielded the following observations. In male adolescents (see Figure 4), there is an age-related broadening of the zygomatic arch, mandible, and bridge of the nose represented in one continuous cluster (DF = 287, p = 3.5*×*10^−7^, *v = *1.95×10^7^ *mm^3^*, peak t-value  = 13.0). Age-related decreases in the local volume are localized (in a single cluster) around the nose, lips, forehead, region of the eyebrow, bottom of the chin and in the temples, lateral to the forehead (DF = 287, p = 5.5*×*10^−7^, v = 1.3×10^7^ *mm^3^*, peak t-value = −21.9).

**Figure 5:**
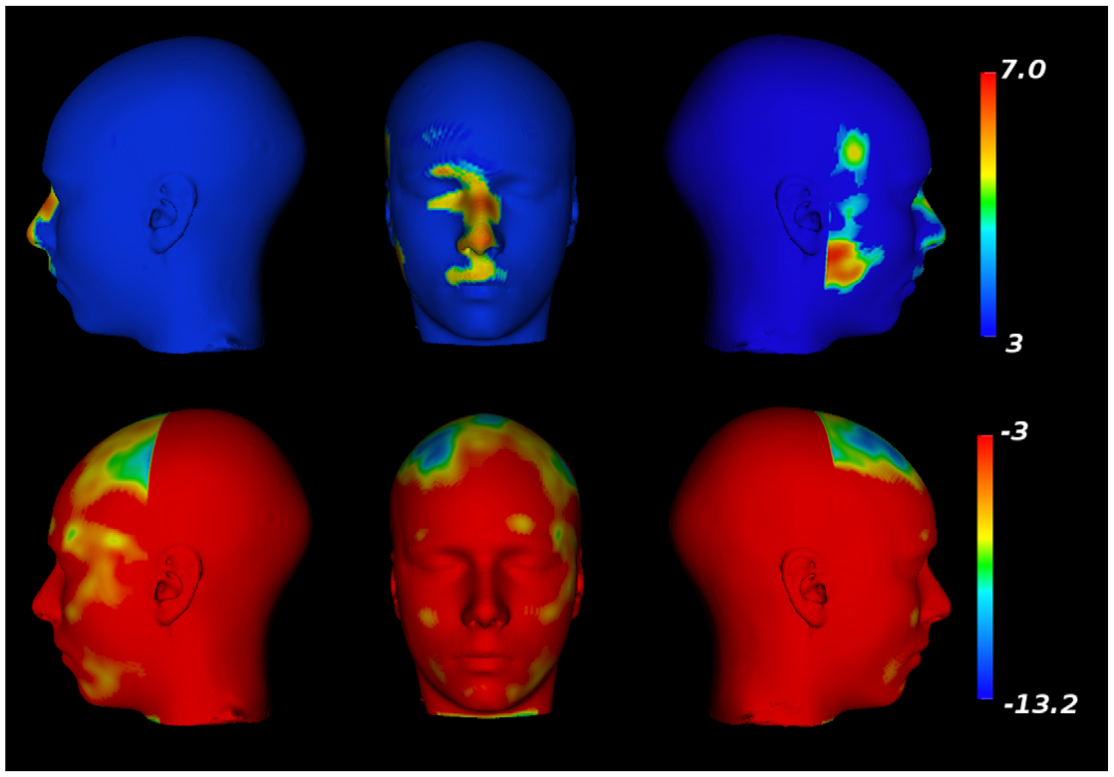
Facial morphometry changes related to age in females. Top row: Facial expansions related to age. Bottom row: Facial contractions related to age.

In female adolescents, there is very focal evidence of age-related changes in the facial structure (see Figure 5). Large age-related changes in the structure of the nose, the filtrum and the lips can be observed (DF = 286, p = 5.8*×*10^−7^, v = 1.7*×*10^6^ *mm^3^*, peak t-value  = 7.0). Similarly, local expansions in the mandible and temple are also observed (DF = 286,p = 5.8*×*10^−7^, v = 3.8*×*10^5^, t-value = −13.2). Age-related decreases in local volumes were found in the region of the scalp directly above the forehead (DF = 286, p = 5.8*×*10^−7^, v = 4.4*×10^5^* *mm^3^*, peak t-value = −13.2). Other age-related decreases are also observed above the eyebrow ridge (DF = 286, p = 4.2×10^−6^, v = 3.5×*10^5^*, peak t-value  = −7.4), left zygomatic arch (DF = 286, p = 0.00012, v = 3.3×10^4^, peak t-value = −6.1), right zygomatic arch (DF = 286, p = 0.00015, v = 3.3×10^4^, peak t-value = −6.4), and mandible (DF = 286, p = 0.0007, v = 3.8×10^4^, peak t-value = −5.8).

### Accuracy and precision of nonlinear transformations

The evaluation of the warped landmarks against the landmarks that were manually placed on 10 individual faces demonstrates great precision in the anatomical localization of specific craniofacial features (see Table 3). For all 17 landmarks the standard deviations of the Euclidean distance were extremely low (maximum standard deviation  = 2.61 mm). Only the standard deviations for the landmarks representing the lateral and medial canthus of the left eye and the bottom the chin exceeded 2 mm. For all other landmarks, the standard deviations of the Euclidean distance were <1.37 mm. The largest differences in Euclidean distance were observed in all four of the landmarks in the eye and the left mouth seam (10.10 to 10.83 mm). Amongst the remaining landmarks, the mean difference in Euclidean distance did not exceed 5.23 mm. For 9/17 landmarks, the mean difference in the distance between landmarks was <3 mm.

### Anthropometric results

The anthropometric indices (see Table 4) estimated for eye length are significantly larger in females, compared with males, for both left (F = 7.12, p<0.0001) and right eyes (F = 3.76, p = 0.0002). There are marginally significant interactions between sex and age for left (F = 2.00, p = 0.046) and right (F = 2.52, p = 0.012) eye height. In both cases, these differences are due to the age-related increase in eye height in the males (F = −2.56, p = 0.011 and F = −2.57, p = 0.011 for left and right sides, respectively). Mouth width also shows significant interactions between age and sex (F = 5.63, p<0.0001) that reflect age-related increases in mouth width in males (F = 8.80, p<0.0001) and females (F = 2.48, p = 0.0135). The distance from ear-to-ear shows a significant interaction between age and sex (F = −3.74, p = 0.002); this is due to age-related increases in males (F = 3.24, p = 0.0013) and decreases in females (F = −2.01, p = 0.0458). The distance between the zygomatic arches is significantly larger in females, compared with males (F = 4.83, p<0.0001). There are significant interactions between age and sex for nose width (F = −5.22, p<0.0001), the filtrum (F = −2.50, p<0.013), the distance between nose and chin (F = −3.01, p = 0.0028), and the bottom of the lip to the chin (F = −2.28, p = 0.0233). The nose width (F = 5.32, p<0.0001), the filtrum length (F = 2.73, p<0.0068), nose and chin length (F = 5.19, p<0.0001), and bottom lip to chin length (F = 5.19, p<0.0001) all show age-related increases in males. Only the nose to chin (F = 1.98, p<0.0487) and bottom lip to chin lengths (F = 2.51, p<0.0125) show significant age-related increases in females.

### Principal components analysis of the PDM

The first 10 PCs in this analysis account for 75.3% of the variability in the distribution of landmark position (see Table 5). PC1 and PC2 account for 46% of the variability in the landmark positions (36.0% and 10.0% respectively). Results from the analysis of PC scores are shown in Table 6. The scores of PC1 show significant interactions between age and sex (F = −4.10, p = 0.0001), mainly due to age-related increases in the PC1 scores of males (F = 5.57, p = 0.0005) but not females (F = 0.04, p = 0.97). The scores for PC2 show no significance with respect to sex but a slight significance with respect to age (F = −2.64, p<0.046). Scores for PC3 and PC4 show significant interactions of age and sex (PC3: p = 0.0005; PC4: p = 0.0006); this is due to significant age-related decreases in the PC3 and PC4 scores of males (PC3: F = −6.02, p<0.0001; PC4: F = −3.4, p = 0.0008) but not females (PC3: F = −1.34, p = 0.18; PC4: F = −1.50, p = 0.13). Scores for PC5 show significant main effects of sex and age (P<0.0001 for both effects).

**Table 5:** Results from the principal component analysis of landmarks representing facial features.

*Principal Component*	*Cumulative Weight (%)*	*Individual Weight (%)*
1	36.0	36.0
2	46.0	10.0
3	53.6	7.7
4	59.3	5.7
5	63.3	4.0
6	66.5	3.2
7	69.0	2.5
8	71.3	2.2
9	73.4	2.1
10	75.3	1.9

**Table 6:** Analysis of subject-wise loading from PCA from the PDM.

*Principal Component*	*Sex*	*Age*	*Age* * *Sex*
1	−1.76	4.23***	−4.10***
2	−0.06	−2.64*	1.93
3	8.40***	−5.04***	3.46**
4	9.26***	−5.21***	3.59**
5	5.01***	−4.70***	0.69

*p<0.05, **p<0.001, ***p<0.0001

In each case, results are summarized as the linear model coefficients (values greater than 0 for sex indicate greater values in females).

**Figure 6:**
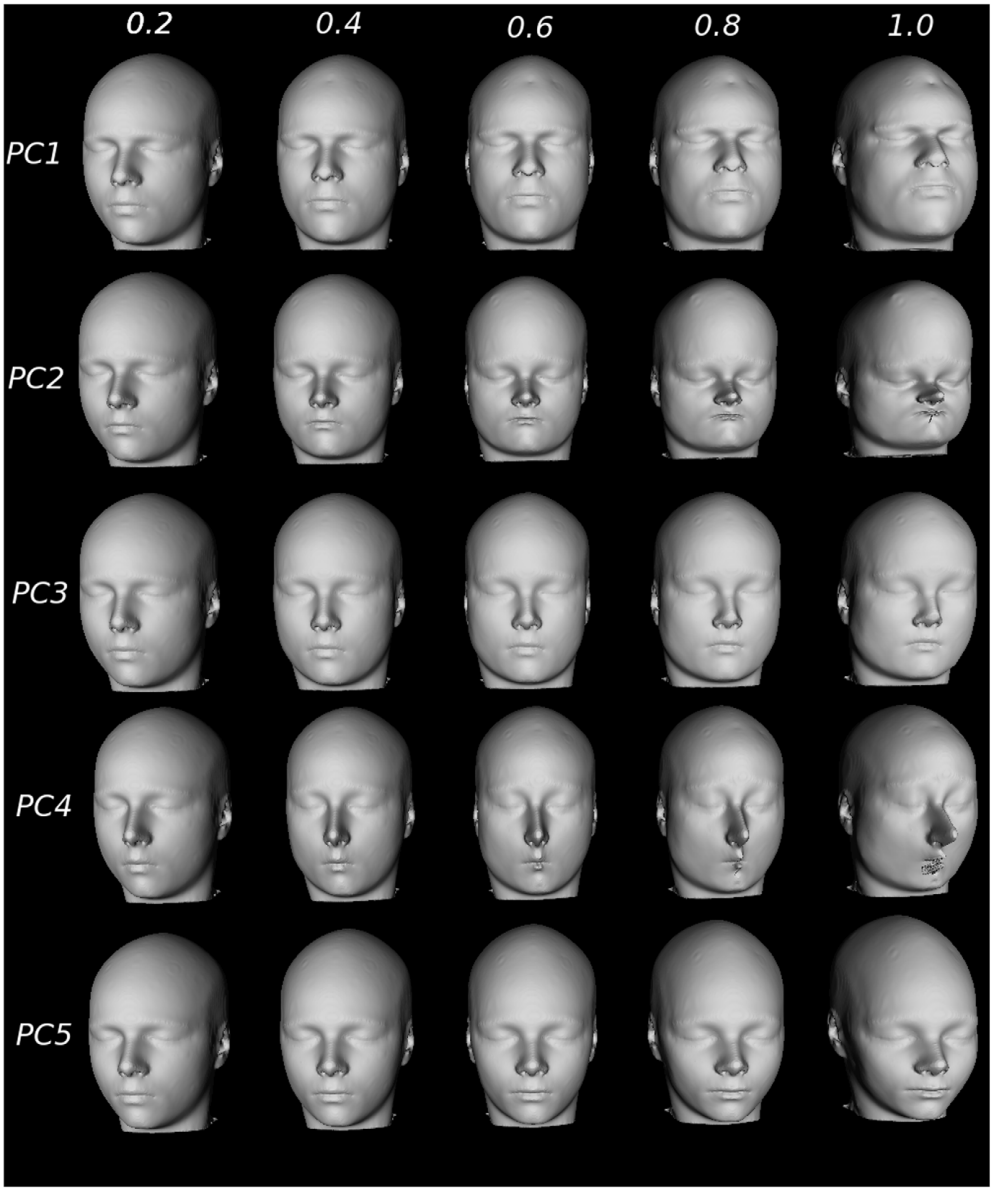
Facial feature simulation created by warping the average face using defined landmarks (see Fig. 5). PCs 1–5 are warped using 0.2, 0.4, 0.6, 0.8, and 1 of each PC score.

**Figure 7:**
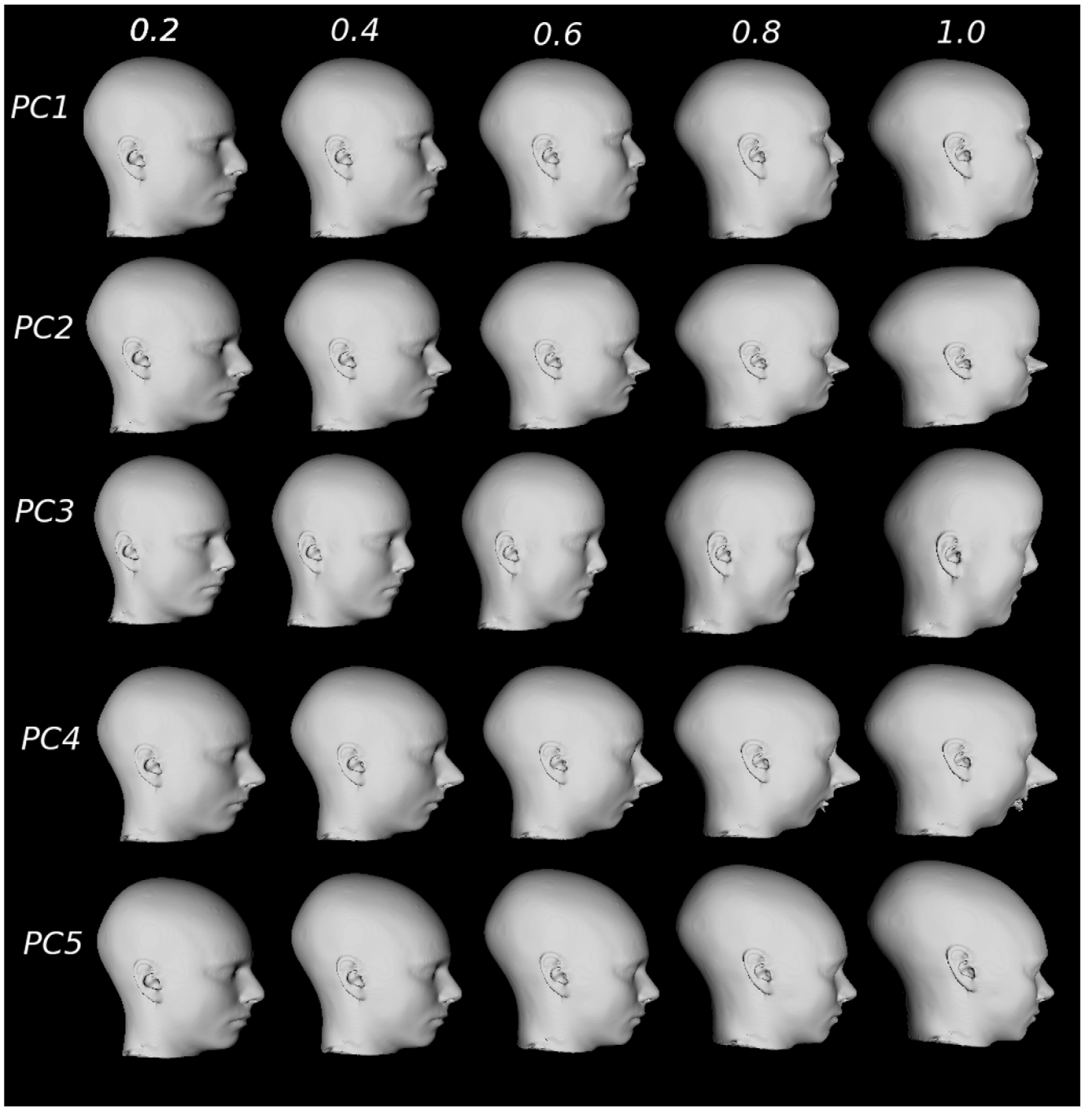
Facial feature simulation (profile view) created by warping the average face using defined landmarks (see Fig. 5). PCs 1–5 are warped using 0.2, 0.4, 0.6, 0.8, and 1 of each PC score.

Results of the simulation are shown in Figure 6 (the entire face) and Figure 7 (profile view). Each simulation demonstrates how each PC encodes a different relationship between facial features. Overall, PC1 demonstrates broadening of the forehead, chin, jaw, and nose; for PC2 the distance between all facial structures decreases and shows an increasing prominence of the forehead; PC3 is characterized by an enlarging brow line, broadening of the zygomatic arch and a less prominent jaw/chin; PC4 is characterized by a broadening of the chin, narrowing of the jaw and mouth, elongation of the nose, and a retreating jawline; and PC5 shows narrower cheekbones, fuller but narrower lips and a less prominent jawline. Note the exaggeration of facial features when the eigenvalue is fully sampled (last column in Figs 6 and 7), thereby providing a simulation of the relationship between different features within the population being studied.

## Discussion

In this paper we have presented a novel methodology for the analysis of craniofacial structure using structural MR images. We demonstrate how techniques originally developed for the image processing and statistical analysis of structural and functional neuroimaging data can be adapted for this purpose. A population-based average of craniofacial structure was estimated using a hierarchical and iterative anatomical matching technique using the head MRI (after removing the brain). The resulting nonlinear transformation matches the craniofacial structure of each subject to the average of the population. Fifty-six landmarks were placed on the average model and warped back to fit each subject using this nonlinear transformation. Voxel-wise analysis shows sexual dimorphism and age-related changes in craniofacial structure. The PDM derived from the landmark-based analysis demonstrates several modes of variation that describe the difference between males and females, and age-related changes during male adolescence. To the best of our knowledge, this is the first demonstration of a fully automated three-dimensional analysis of craniofacial structure using MRI data.

### Significance of the findings

Our voxel-wise deformation analysis shows clear differences between male and female adolescents (Fig. 2). The broadening of the chin, zygomatic arch, and forehead are consistent with work demonstrating changes in the facial features of young men in relationship with salivary testosterone [13]. Compression of the nose relative to the face is also consistent with previous reports of sexual dimorphism in nose structure [48]. Age-related changes in male adolescents demonstrate that some of these findings may be related to changes in facial features during maturation. Compression of the temple may be indicative of a broadening of the brow line that occurs during male maturation caused by a surge in testosterone levels [13]. Female adolescents demonstrate few changes in the way of facial features as a function of age. Since growth spurts occur earlier in girls than boys [49], much of the age-related changes in facial features could have occurred (in girls) at an earlier age (i.e. outside the 12-to-18 age range of this cohort). There may be a similar reason for the lack of local age-related compressions in craniofacial features observed in the voxel-wise analysis.

The anthropometric results demonstrate similar changes in the size and shape of the eyes, size of the chin, and mouth. These results do not, however, capture the relationship between any different facial features. On the other hand, the results from the PCA analysis from PDM allow us to understand better such spatial relationships. Our results from PC1 and PC3 demonstrate broadening of similar areas of the jaw, zygomatic arch, and browline. Age-related changes in subject-wise loadings on PC1 and PC3 in male adolescents are going in the opposite directions: negative for PC1 and positive for PC3. Clearly, two different biological processes are at play here; future studies may help us understand whether, for example, “masculinization” (PC1) and “demasculinization” (PC3) of the facial features could be related to the balance of male and female sex hormones. PC5 clearly demonstrates higher values in females. This PC mimics the results in shape regression against the 2D:4D ratio [12], [13] in young men. Increased 2D:4D ratio, possibly indicating lower exposure to fetal testosterone, was associated with a narrower mouth width, larger distance between the nose and mouth, and fuller lips. Lower prenatal and salivary testosterone also showed significant association with nose structure [12], [13]. We see that female traits are strongly associated with elongation and narrowing of the nose (similar findings have been reported elsewhere [48]) and changes in the position of the chin (see Figs 6 and 7). Although Fink and colleagues [12], [13] showed a narrowing and broadening of the chin with respect to high and low 2D:4D ratios, respectively, it is possible that these changes demonstrate differences in anterior-posterior chin position, which our analyses show explicitly.

### Choice of image modality

Most biomedical research of craniofacial morphology has used photographs and advanced laser-scanning techniques to image the exterior of the face. Since we have used MR images here, we are able to take into account three-dimensional representations of the face. Like previous studies, however, the analysis presented does not dissociate soft tissues (fat and skin) from the skull. Thus, all measures provided are a composite of measures of the skull and soft tissues and the actual source of craniofacial features detected here cannot be identified. This could be addressed in analyses where the skull is segmented from the MRI data [38] or by using computer-assisted tomography for skull imaging, as has been done in transgenic mice [50]. To analyze local percentages of body fat, novel techniques would have to be derived for the segmentation of fat in the human face.

In light of these limitations and to test the robustness of our findings, we performed a second analysis where percent body-fat was accounted for using the residual error after regression of each PC score against total body-fat assessed with bioimpedance, a standard measurement in the SYS data acquisition protocol [25]). Results of these analyses are shown in Table S2 and are similar for PCs 3, 4 and 5 as presented in Table 6. Removing the effect of total fat increased the significance of the sex by age interaction and the effect of sex observed for PC1. Residuals of PC2 now show significant, albeit subtle, differences with respect to sex (p = 0.048) and interactions of age and sex (p<0.0004). These supplementary findings suggest that a measure of fat (and potentially other measures of body composition) may be useful when analyzing the variability of face morphology with surface-based techniques, such as photographs, laser scanning or MRI.

### Choice of image analysis technique

Anthropological work on the evolutionary changes of the cranium has used three-dimensional PDMs [2]. In our work, we matched craniofacial structure using image-intensity features; the creation of a PDM is, thus, simplified as the initial landmarks need only be defined once on the population average. Similarly, the work of Hennessy et al. [9], [14] used three dimensional laser scanning technology for the analysis of the facial exterior in the context of sexual dimorphisms [14]. In their work, gross geometric changes were analyzed using PDMs derived from the identification of manually placed landmarks. A similar approach was used in the analysis of facial features in patients suffering from schizophrenia using T1-weighted MRI data [8], [15]. Our methodology could be used in MR cohorts with larger numbers of participants where the information on craniofacial structure is available. Similarly, there are possible applications for this work in other disorders such as Pierre Robbin [51], Down Syndrome [52], Fetal Alcohol Syndrome [53], and others [54].

Deformation-based analyses have created some controversy in the neuroimaging literature due to their inability to match differing gyrification patterns between individuals [55]. This approach is, however, ideal for the analysis of craniofacial structure as homologous anatomy (e.g. eyes, nose, mouth) is present in almost all individuals. Since parameters optimized for neuroimaging data were used for the estimation of nonlinear transformations, it is possible that the nonlinear transformations estimated were suboptimal. The approach used for averaging used eight different levels of resolution (where the blurring kernel and the spacing between the local translation estimated decreases at each successive iteration; see Table 2) and the changes in craniofacial structure are not morphologically complex. Optimizations of ANIMAL used for histological data [56] suggest that parameter choice may be dependent on image contrast and not entirely on structure. For example, the regularization parameter used for deformation-based morphology using MRI data from the mouse [33], [34] are similar to those optimized for human MRI data [41]. Nonetheless, optimization of nonlinear registration techniques for analyses of craniofacial structure presents a different challenge in comparison with neuroimaging, and will likely require exhaustive analyses similar to those previously presented in the brain nonlinear registration literature [57], [58], [59]. Each of these studies presents challenges in the definition of a “gold-standard” used for comparison and optimization [44], [57]. Our own analysis of the accuracy of the nonlinear transformations demonstrates very high precision between the automatically and manually defined landmarks. For 5 of these landmarks, however, we see some discordance between the two sets of landmarks. This may reflect poor accuracy of the nonlinear deformations in this particular region of the face. But given the high level of repeatability of the nonlinear transformations, we feel that this is unlikely. Since the manual rater did not have the benefit of a three-dimensional surface (like the one used to define the initial landmarks on the average face), this discrepancy might reflect a systematic difference between the two landmarking methodologies. Moreover, it underscores a need for robust automated techniques for defining craniofacial landmarks.

The atlas-building strategy may also require further investigation. A large field of research suggests that there are optimal methods for the creation of an atlas that best represents the group being studied. Some of these methods involve the simultaneous estimation of transformations that warp all subjects to a group average in an iterative unbiased fashion [60] using large deformation diffeomorphic template estimation techniques [61], [62].

While our PDM uses 56 landmarks, the number of landmarks can be increased by creating an equally spaced grid over the entire craniofacial structure, similar to the work done in the analysis of the hominoid cranium [63]. This type of analysis would limit bias but it would increase the computational complexity and dimensionality of the analysis. Similarly, the total number of anatomically localized landmarks could also be increased.

Unlike previous methodologies, our method can analyze the entire ensemble of facial features using voxel-wise analyses and simulations are only limited to the number of landmarks chosen. Further, more sophisticated models of shape analysis could be used to analyze the modes of variance directly from the deformation fields, such as an active appearance model [64] which has been previously used in the computer aided diagnosis of different forms of Alzheimer's disease and dementia [65], [66].

## Supporting Information

Figure S1 **Population distribution for subjects used in this study for (A) males and (B) females.**
(PNG)

Table S1 **Full description of all landmarks used for analysis of facial morphometry.**
(DOC)

Table S2 **Analysis of subject-wise loading from PCA from the PDM of facial features on the residuals from regression of PCs against percent body fat.**
(DOC)
