# Supplementary figures and images for: Automated Analysis of Craniofacial Morphology Using Magnetic Resonance Images
Source: PLoS One. 2011 May 31;6(5):e20241. doi: 10.1371/journal.pone.0020241 (PMC3105012; doi:10.1371/journal.pone.0020241)

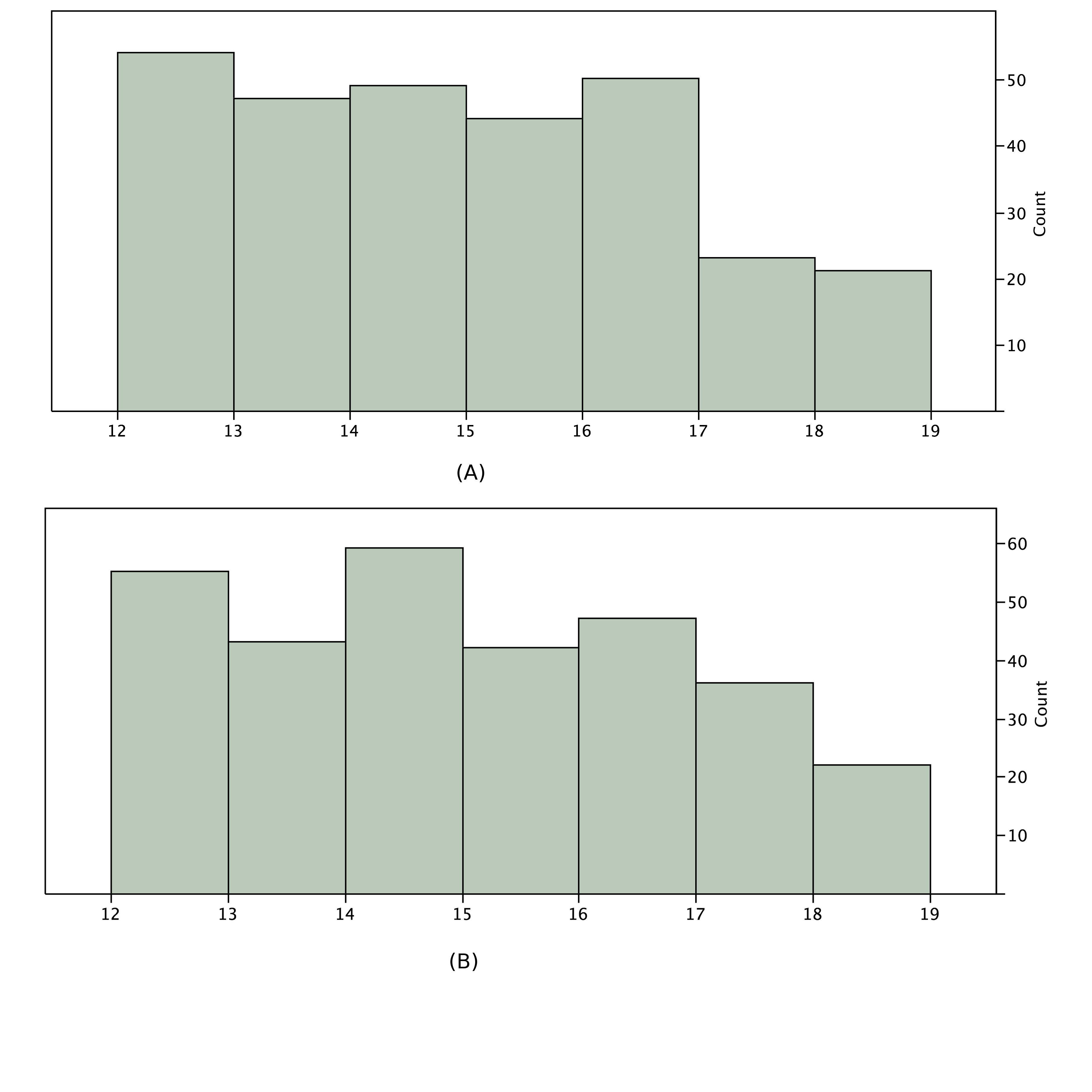

Supplement: Figure S1 — Population distribution for subjects used in this study for (A) males and (B) females. (PNG) [file pone.0020241.s001.png]
