# Supplementary material for: Automated Analysis of Craniofacial Morphology Using Magnetic Resonance Images
Source: PLoS One. 2011 May 31;6(5):e20241. doi: 10.1371/journal.pone.0020241 (PMC3105012; doi:10.1371/journal.pone.0020241)
Supplement: Table S1 — Full description of all landmarks used for analysis of facial morphometry. (DOC) [file pone.0020241.s002.doc]

Table S1 Full description of all landmarks used for analysis of facial morphometry.

| **Landmark Number** | **Label** | **Definition** |
| --- | --- | --- |
| 1 | Right Hairline | identify the Middle Hairline point (#2) first and then mark the Right and Left Hairline points (#1, #3) equidistant along the x-axis; all points should have comparable z-plane coordinates |
| 2 | Middle Hairline |
| 3 | Left Hairline |
| 4 | Lateral Right Eyebrow | identify the Lateral and Medial Eyebrow points; calculate the x-coordinate of the mid-point based upon 1/2 of the distance between the lateral and medial points (which is added to the x-coordinate of the medial x coordinate) |
| 5 | Mid Right Eyebrow |
| 6 | Medial Right Eyebrow |
| 7 | Medial Left Eyebrow | identify the Lateral and Medial Eyebrow points; calculate the x-coordinate of the mid-point based upon 1/2 of the distance between the lateral and medial points (which is subtracted from the x-coordinate of the medial x coordinate) |
| 8 | Mid Left Eyebrow |
| 9 | Lateral Left Eyebrow |
| 10 | Lateral Right Eye | lateral edge of the right eye |
| 11 | Medial Right Eye | medial edge of the right eye |
| 12 | Upper Apex Right Eye Orbit | maximal upper apex of the right eye orbit |
| 13 | Mid Apex Right Eye Orbit | middle apex of the right eye orbit (along the lower eyelid) |
| 14 | Lower Apex Right Eye Orbit | maximal lower apex of the right eye orbit |
| 15 | Medial Left Eye | medial edge of the left eye |
| 16 | Lateral Left Eye | lateral edge of the left eye |
| 17 | Upper Apex Left Eye Orbit | maximal upper apex of the left eye orbit |
| 18 | Mid Apex Left Eye Orbit | middle apex of the left eye orbit (along the lower eyelid) |
| 19 | Lower Apex Left Eye Orbit | maximal lower apex of the left eye orbit |
| 20 | Middle Base of the Nose | all points should have comparable x-coordinates |
| 21 | Tip of the Nose |
| 22 | Bridge of the Nose |
| 23 | Upper Right Nostril | top of the nostril; medial point; flush against the face surface |
| 24 | Upper Left Nostril | top of the nostril; medial point; flush against the face surface |
| 25 | Mid Right Nostril | farthest lateral peak of the nostril |
| 26 | Mid Left Nostril | farthest lateral peak of the nostril |
| 27 | Lower Right Nostril | anterior peak of the inside surface of the nostril |
| 28 | Lower Left Nostril | anterior peak of the inside surface of the nostril |
| 29 | Right Corner of Mouth | lateral corner of the mouth |
| 30 | Right Mid-Upper Lip | x-coordinate is 1/2 between 29 & 31 |
| 31 | Right Superior Peak of the Upper Lip | superior peak of the lip |
| 32 | Inferior Peak of the Midpoint of the Upper Lip | inferior peak of the midpoint of the upper lip; x-coordinate should be approximately 1/2 between 31 & 33; 29 & 35 |
| 33 | Left Superior Peak of the Upper Lip | superior peak of the lip |
| 34 | Left Mid-Upper Lip | x-coordinate is 1/2 between 33 & 35 |
| 35 | Left Corner of Mouth | laternal corner of the mouth |
| 36 | Right Mid-Lower Lip | x-coordinate is 1/2 between 29 and 37; on the lip surface |
| 37 | Inferior Peak of the Lower Lip | inferior peak of the lower lip; on the lip surface |
| 38 | Left Mid-Lower Lip | x-coordinate is 1/2 between 35 and 37; on the lip surface |
| 39 | Right Mid-Mouth Seam | x-coordinate is 1/2 between 29 and 40 |
| 40 | Mid-Mouth Seam | mid-point of the seam of the lips; approximately same x-coordinate as 32 & 37 |
| 41 | Left Mid-Mouth Seam | x-coordinate is 1/2 between 35 and 40 |
| 42 | Right Ear | bottom-most point of the ear trough |
| 43 | Left Ear | bottom-most point of the ear trough; z co-ordinate should be inn line with 42 |
| 44 | Bottom of Chin | maximum curvature of the chin along the jawline |
| 45 | Right Mid-Jaw | on the jawline; 1/2 the z-coordinate distance between 42 & 44 |
| 46 | Left Mid-Jaw | on the jawline; 1/2 the z-coordinate distance between 43 & 44 |
| 47 | Right Upper Quadrant - Jaw | on the jawline; 1/2 the z-coordinate distance between 42 & 45 |
| 48 | Left Upper Quadrant - Jaw | on the jawline; 1/2 the z-coordinate distance between 43 & 46 |
| 49 | Right Lower Quadrant - Jaw | on the jawline; 1/2 the z-coordinate distance between 44 & 45 |
| 50 | Left Lower Quadrant - Jaw | on the jawline; 1/2 the z-coordinate distance between 44 & 46 |
| 51 | Right Lower Eigth - Jaw | on the jawline; 1/2 the z-coordinate distance between 44 & 49 |
| 52 | Left Lower Eigth - Jaw | on the jawline; 1/2 the z-coordinate distance between 44 & 50 |
| 53 | Right zygomatic arch | Below right eye and end of posterior curvature of the zygomatic arch |
| 54 | Right – anterior zygomatic arch | Anterior portion of the zygomatic arch at the apex of the anterior curvature |
| 55 | Left zygomatic arch | Below left eye and end of posterior curvature of the zygomatic arch |
| 56 | Left – anterior zygomatic arch | Anterior portion of the zygomatic arch at the apex of the anterior curvature |
