# Supplementary material for: Automated Analysis of Craniofacial Morphology Using Magnetic Resonance Images
Source: PLoS One. 2011 May 31;6(5):e20241. doi: 10.1371/journal.pone.0020241 (PMC3105012; doi:10.1371/journal.pone.0020241)
Supplement: Table S2 — Analysis of subject-wise loading from PCA from the PDM of facial features on the residuals from regression of PCs against percent body fat. (DOC) [file pone.0020241.s003.doc]

Table S2 Analysis of subject-wise loading from PCA from the PDM of facial features on the residuals from regression of PCs against percent body fat. In each case results are summarized as the linear model coefficients (values greater than 0 for sex indicate greater values in females).

| ***Principal Component*** | ***Sex*** | ***Age*** | ***Age*Sex*** |
| --- | --- | --- | --- |
| 1 | -6.19*** | 0.46 | -5.40** |
| 2 | 2.06* | -0.41 | 2.04* |
| 3 | 7.61*** | -5.57*** | 3.17* |
| 4 | 9.73*** | -5.57*** | 3.61** |
| 5 | 5.88*** | -3.80*** | 0.86 |

*p<0.05, **p<0.001, ***p<0.0001
